# Streptococcal superantigens and the return of scarlet fever

**DOI:** 10.1371/journal.ppat.1010097

**Published:** 2021-12-30

**Authors:** Jacklyn R. Hurst, Stephan Brouwer, Mark J. Walker, John K. McCormick

**Affiliations:** 1 Department of Microbiology and Immunology, Schulich School of Medicine & Dentistry, University of Western Ontario, London, Ontario, Canada; 2 Australian Infectious Diseases Research Centre, School of Chemistry and Molecular Biosciences, University of Queensland, St. Lucia, Queensland, Australia; 3 Lawson Health Research Institute, London, Ontario, Canada; Nanyang Technological University, SINGAPORE

## Abstract

*Streptococcus pyogenes* (group A *Streptococcus*) is a globally disseminated and human-adapted bacterial pathogen that causes a wide range of infections, including scarlet fever. Scarlet fever is a toxin-mediated disease characterized by the formation of an erythematous, sandpaper-like rash that typically occurs in children aged 5 to 15. This infectious disease is caused by toxins called superantigens, a family of highly potent immunomodulators. Although scarlet fever had largely declined in both prevalence and severity since the late 19th century, outbreaks have now reemerged in multiple geographical regions over the past decade. Here, we review recent findings that address the role of superantigens in promoting a fitness advantage for *S*. *pyogenes* within human populations and discuss how superantigens may be suitable targets for vaccination strategies.

## The discovery of scarlet fever toxins

Early in the 20th century, American bacteriologists Gladys and George Dick induced experimental scarlet fever in human volunteers through the inoculation of hemolytic streptococci that had been isolated from a nurse’s finger lesion after caring for a convalescent scarlet fever patient. From these experiments, they concluded that *Streptococcus pyogenes* was the etiological cause of scarlet fever [1]. The classic scarlet fever rash, characterized as a finely papular erythematous rash and a so-called “strawberry tongue,” was also induced by injecting volunteers intradermally with sterile supernatant filtrates from scarlet fever isolates, revealing the “scarlet fever toxin.” This procedure was termed the “Dick test” and was subsequently used to identify individuals susceptible to developing scarlet fever through the appearance of a local hypersensitive reaction to the toxin-containing filtrate [1]. Over a century later, it is now clear that scarlet fever toxins, also commonly referred to as streptococcal pyrogenic exotoxins (Spes), are part of a larger family of immunostimulatory bacterial proteins known as superantigens. Typically, in the context of severe disease, superantigen activity can lead to the potent and broad activation of numerous T cells leading to a devastating cytokine storm-mediated disease known as the toxic shock syndrome [2]. Yet, how these toxins may contribute to the life cycle of *S*. *pyogenes*, and why they are encoded with such apparent redundancy in the *S*. *pyogenes* genome, is only now beginning to be understood.

## Resurgence of epidemic scarlet fever

Scarlet fever is a disease of historic significance, described as early as around 400 BC by Hippocrates, and reports during the premodern period (1,500 to 1,900 CE) often describe scarlet fever epidemics with very high mortality rates [3]. Although the incidence of scarlet fever largely declined in both frequency and severity during the 20th century, the exact cause(s) for the progressive decline in scarlet fever remain largely unknown, which began prior to the widespread use of antibiotics [4]. It has been suggested that improved dietary standards and public health measures may have contributed to the observed decrease in prevalence of scarlet fever [5–7]. However, an unexpected surge in cases has been reported since around 2011 in mainland China, Hong Kong, South Korea, Taiwan, Singapore, and Vietnam, as well as in the United Kingdom and other European countries since around 2014 [8].

Epidemiological surveillance studies have shown that multiclonal *emm12* and *emm1* lineages were the predominant scarlet fever-associated streptococcal *emm*-types circulating in Hong Kong, China, and Taiwan during the 2011 outbreak [9–11]. The majority of scarlet fever-associated lineages contained the unifying characteristic of 2 important mobile genetic elements: toxin-harboring prophages encoding streptococcal superantigens SSA and SpeC, and the DNase Spd1 (e.g., ΦHKU.vir), and integrative and conjugative elements (ICEs) encoding multidrug resistance to tetracycline and macrolides (e.g., ICE-*emm12*) [9,12]. Although these strains were already circulating in the regional population in the years preceding the 2011 outbreak [9], increased antibiotic usage and resistance may have conferred selection and expansion of these strains over time [13]. Epidemiological surveillance from the UK scarlet fever outbreak in 2014 determined that regional outbreaks were caused by multiple *emm3*, *emm12*, *emm1*, and *emm4* types and phylogenetic lineages, and significant increased prevalence of a single gene, *ssa*, was associated with scarlet fever cases [14]. In 2019, emergence of a novel dominant clone of the *emm1* genetic lineage designated “M1_UK_” was first reported in the UK and associated with seasonal surges of scarlet fever and a marked increase in invasive infections [7]. The genomic structure of the M1_UK_ lineage revealed the accumulation of a series of 27 single nucleotide polymorphisms that caused a markedly enhanced production of the SpeA superantigen (approximately 10-fold) compared to classic M1T1 isolates [7].

## Superantigen contribution to host colonization by *S*. *pyogenes*

Scarlet fever often presents with a fine red rash in both adults and children. The characteristic erythrogenic rash was originally considered as a result of primary toxicity; however, it is now understood to be a result of a delayed host-acquired hypersensitive reactivity to streptococcal antigens. As the development of the rash is not observed among those with no prior streptococcal infection [15], previous exposure to *S*. *pyogenes* likely contributes to the amplified hypersensitive reaction. The enhanced skin reactivity likely results from the rapid and extensive cytokine release and infiltration of leukocytes from secondary antigen exposure that is amplified by the lymphocyte mitogenicity induced by superantigens. Animals injected with superantigens demonstrate no scarlet fever rash, yet if animals are presensitized with streptococcal antigens or superantigens, a rash occurs with subsequent intradermal challenge [15].

**Fig 1:**
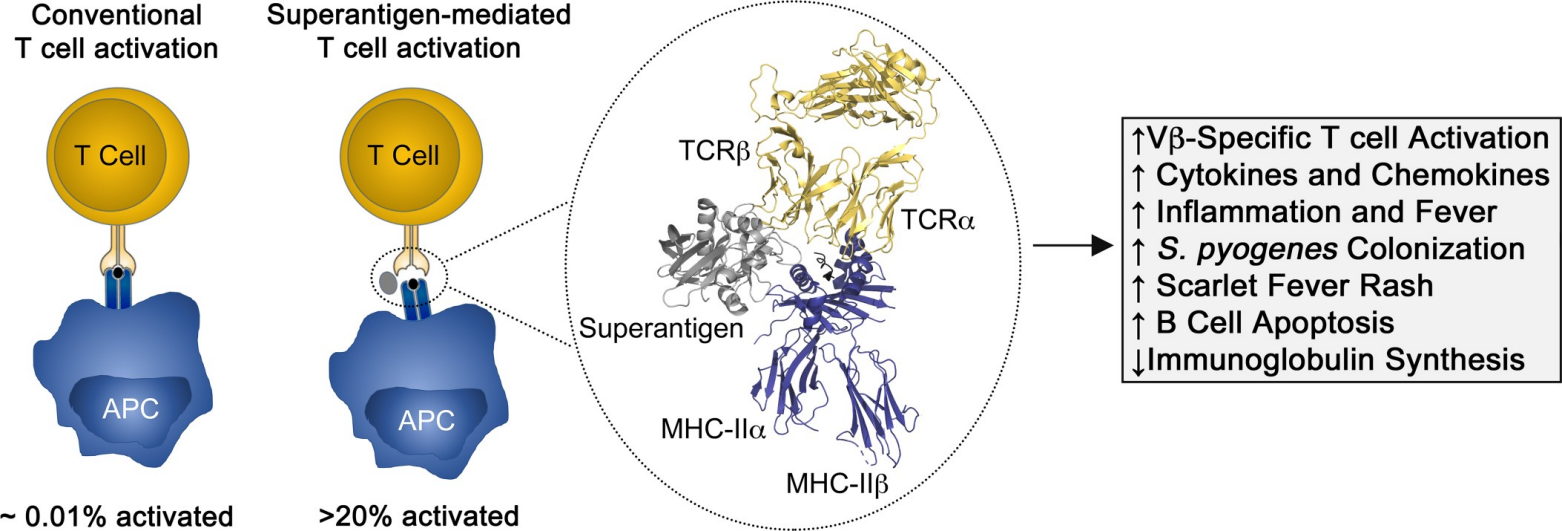
Overview of T cell activation by streptococcal superantigens and functional outcomes. Displayed are cartoons showing a comparison of conventional versus superantigen-mediated T cell activation, and a ribbon diagram of the SpeA superantigen (gray) in complex with the TCR expressed on a T cell (yellow) and MHC-II expressed on an APC (blue). Compared with convention T cell activation, note how the superantigen engages the β-chain of the TCR and displaces the TCR away from the MHC-II presented antigenic peptide (black) leading to the Vβ-specific activation of T cells. The structural model was generated using the co-crystal structure of SpeA in complex with mouse Vβ8 (PDB code 1L0Y) and superimposing a human αβ TCR (PDB code 1FYT) and human MHC-II from the staphylococcal enterotoxin C3 superantigen in complex with HLA-DR1 (PDB code 1JWM). The ribbon diagram was generated using PyMOL Molecular Graphics System (https://pymol.org). APC, antigen presenting cell; MHC-II, MHC class II molecule; TCR, T cell receptor.

Mechanistically, superantigens function to “force” the broad activation of a large number of T cells by bridging the V region of TCR β-chains with MHC class II molecules (MHC-II) (**Fig 1**). This unusual Vβ-specific activation of T cells differs from “classical” T cell activation because superantigens bypass prior processing and presentation, and such, the peptide specificity of the T cell becomes irrelevant. Yet, it has long remained enigmatic as to why *S*. *pyogenes* would encode and produce multiple superantigens that cause excessive activation of the adaptive immune system and how this benefits bacterial fitness and host colonization.

To more accurately reflect the strict human tropism of *S*. *pyogenes*, transgenic mice that express human leukocyte antigen (HLA) MHC-II molecules have been used to both study superantigen biology and model *S*. *pyogenes* infection [16]. Using HLA-DQ8 expressing transgenic mice in a nasal infection model, we previously established that SpeA can dramatically enhance the ability of the rheumatic fever isolate *S*. *pyogenes* MGAS8232 to cause acute, upper respiratory tract infection [17]. In absence of a functional SpeA superantigen, an appropriate human MHC-II receptor, or functional Vβ-specific T cells, *S*. *pyogenes* MGAS8232 is unable to colonize the nasopharynx and multiply in this model [18]. These findings thus provide a conceptual framework to explain the emergence and subsequent dominance of the novel M1_UK_ clone within the population of upper respiratory tract isolates from the UK [7]. Additionally, we recently demonstrated that acquisition of the ΦHKU.vir prophage encoding the superantigen SpeC and the DNase Spd1 promoted nasopharyngeal colonization fitness of a Hong Kong scarlet fever isolate *S*. *pyogenes* HKU16 in HLA-transgenic mice [19]. The precise role for the superantigen SSA in the etiology of scarlet fever, also detected in the majority of Hong Kong scarlet fever isolates [9,12], however, remains undefined. Since SSA is unique among the streptococcal superantigens in that it is a thiol-activated toxin, its activity appears to be dependent on host cell and tissue injury [19], indicating a later role for SSA during *S*. *pyogenes* infection. Using the invasive *S*. *pyogenes* M89 serotype SN1, superantigen-sensitive HLA-transgenic mice were also shown to be more susceptible to both invasive (intraperitoneal) and skin infection [20].

Genomic analyses have revealed that the *speA* gene had almost disappeared from M1 isolates obtained in the mid-20th century (1920s to 1980s), which coincided with a sharp decline in severe invasive GAS infections during that time [21]. We can only speculate that this event may have also played a role in the fall of scarlet fever incidence. However, the *spe*A2 allele was introduced in the globally disseminated M1T1 clone in the 1980s, well before the resurgence of scarlet fever (approximately 30 years). Nonetheless, there is increasing evidence that acquisition of prophage carrying genes encoding virulence factors (SpeA, SSA, SpeC, and Spd1) and antibiotic resistance contributes to the increased fitness and virulence of contemporary GAS strains causing scarlet fever and invasive disease [7,9,12,17–19,22]. Collectively, these above studies have established an important role for superantigens in host–pathogen interactions to promote *S*. *pyogenes* infection.

## T cell activation as a mechanism to avoid host immune clearance

By targeting T cells, superantigens stimulate uncontrolled cytokine responses that simultaneously recruit and then subvert the activity of effector cells, impeding the host’s ability to combat infections. Human tonsils exposed to superantigen, including *S*. *pyogenes* supernatants, demonstrate B cell apoptosis with a marked reduction in immunoglobulin synthesis [23]. Furthermore, impaired antibody responses against *S*. *pyogenes* have been identified in patients with recurrent streptococcal tonsillitis. Patients displayed significantly reduced antibody titers against SpeA, SpeA perturbation of germinal center T follicular helper cell populations, and SpeA-induced killing of germinal center B cells through aberrant granzyme B and perforin expression [24]. Different MHC-II alleles were further identified as being either risk-associated or protective for recurrent streptococcal tonsillitis [24]. These findings indicate that superantigens not only promote infection but may also provoke host susceptibility to subsequent infection. The recent findings that select streptococcal superantigens can potently activate mucosal-associated invariant T (MAIT) cells, both directly and indirectly, requires further study in this area [25].

Though the apparent redundancy of *S*. *pyogenes* superantigens has not been entirely explained, antigenic diversity within this toxin family [17] may allow for superantigens to both escape humoral immunity [18] but also to recognize an assorted range of TCR β-chains and MHC-II, thereby subverting initiation of adaptive immune responses (**Fig 1**).

## Are superantigens suitable vaccine candidates?

Humans are the only natural host and reservoir for *S*. *pyogenes*. This human-opportunistic pathogen is thought to exist primarily as an asymptomatic commensal in the upper respiratory tract and skin; therefore, preventing *S*. *pyogenes* carriage is a major goal to prevent all types of *S*. *pyogenes* disease. Substantial vaccine research against *S*. *pyogenes* has targeted the surface-anchored M protein but has faced multiple obstacles that have also hindered development of a commercial vaccine, including antigenic variation, genetic diversity among strains, differential distribution of serotypes globally, and earlier vaccine safety concerns due to the potential for M protein antigens to trigger autoimmune sequelae [26]. Thus, a universally protective vaccine based on this surface molecule remains challenging. Recently, a pharyngeal infection model using nonhuman primates (NHPs) tested the non-M protein–based vaccine candidate, Combo5, containing 5 largely conserved *S*. *pyogenes* protein antigens (arginine deiminase, C5a peptidase, streptolysin O, interleukin-8 protease, and trigger factor). Although Combo5 was not protective against *S*. *pyogenes* colonization, the vaccine did stimulate antibody responses and reduced both pharyngitis and tonsillitis scores [27], highlighting the important use of NHP pharyngeal infection models for assessing *S*. *pyogenes* vaccines.

Although superantigens are usually immunologically distinct [17] and often encoded on mobile prophage elements, the *S*. *pyogenes* superantigen “repertoire” appears to be limited to 13 distinct superantigens [28,29]. Since prophage elements are primary vehicles of exotoxin dissemination, different strains of *S*. *pyogenes* often encode a variable combination of distinct functional superantigens (SpeA, SpeC, SpeG, etc.). As superantigens activate T cells in a TCR Vβ-specific manner, resulting in a “Vβ-skewing” fingerprint, further characterization of TCR Vβ lymphocyte populations in scarlet fever patients may provide greater insight of potential superantigen activity following streptococcal infection. Clear Vβ expansion that is not clonal (i.e., not antigen-specific) would be consistent with specific superantigen-driven responses and may help identify other superantigens that may be active in a particular scarlet fever outbreak.

Although vaccination with inactive toxoid mutants of SpeA and SpeC superantigens show clear protective outcomes in rabbit models of streptococcal toxic shock syndrome [30,31], and a SpeA toxoid vaccine can be protective against experimental nasopharyngeal infection [17], sequencing of 2,083 *S*. *pyogenes* genomes revealed that SpeA and SpeC carriage remains low at <20% and <40%, respectively, in the global *S*. *pyogenes* population [32]. As *S*. *pyogenes* remains a highly genetically distinct species, specific superantigens alone are likely unsuitable vaccine targets for global *S*. *pyogenes* coverage; however, we believe that informed selection of detoxified superantigens should receive renewed consideration for inclusion within a multicomponent vaccine against lineage-specific epidemics of *S*. *pyogenes* to prevent the spread and further resurgence of scarlet fever outbreaks.
